# Comparison of the deformity reduction device (DRD) mini and the Slocum jig in the precision of torsional correction during distal femoral osteotomies in small to medium breed dogs

**DOI:** 10.1371/journal.pone.0308764

**Published:** 2024-09-16

**Authors:** Stephanie K. Steuri, Cassio Ferrigno, Adrien Hespel, Xiaojuan Zhu

**Affiliations:** 1 Department of Small Animal Clinical Sciences, University of Tennessee College of Veterinary Medicine, Knoxville, Tennessee, United States of America; 2 Perth Veterinary Specialists, Osborne Park, WA, Australia; 3 Office of Innovative Technologies, University of Tennessee, Knoxville, Tennessee, United States of America; Sinai Hospital of Baltimore, UNITED STATES OF AMERICA

## Abstract

Previous studies have compared the use of the Slocum- jig to the deformity reduction device (DRD) jig for the correction of distal femoral deformities of varying degrees in the frontal plane. The objective of the current study is to further investigate the use of the mini DRD jig in comparison to the Slocum jig for correction of varying degrees of torsional deformities of the distal femur. Femoral models (n = 60) were developed based on a CT scan of an approximately 16.5 kg normal canine femur. Models were created with a standard varus deformity of 15 degrees, and external torsional deformities of 15, 20, or 30 degrees. Using center of rotation of angulation (CORA) methodology, corrective osteotomies were planned and performed on each of the 3D printed models based on the group assigned. Modeling clay was applied the proximal femur to mimic visualization of a routine lateral surgical approach, while retaining the ability to palpate surgical landmarks. Post-correction anatomic lateral distal femoral angle (aLDFA) and femoral torsion angle (FTA) were measured and recorded. The mean post-correction FTA of groups using the DRD jig for correction were consistently closer to the ideal value compared to those using the Slocum jig, although these differences were not always statistically significant. Correction of external torsion between the DRD jig and the Slocum jig was significantly different in groups 1 and 2 (P = 0.026 and P = 0.046), but not in group 3. For the correction of varus deformity, no significant difference was found between the two jig types in any group. Results of this study suggest jig selection during distal femoral osteotomy for correction of torsional deformity may result in varying precision of post-correction alignment. Clinical significance of this variance remains unclear, and intra-operative visual assessment of alignment should be implemented to guide corrections.

## Introduction

The distal femoral osteotomy is a surgical procedure that can be used to correct patellar luxations that result from significant femoral deformities [1–4]. According to current literature, femoral malformations that include varus deformities greater than 10–12˚ or an anatomical lateral distal femoral angle (aLDFA) greater than 102˚ may require significant realignment surgery, although these cut-off values remain controversial [1–3, 5–8]. Reported recurrence rates following surgical correction of medial patella luxation (MPL) range from 6–36%, with higher assigned grades of preoperative luxation being associated with a greater risk [3, 9]. The use of a distal femoral opening wedge or closing wedge ostectomy has been shown to successfully improve overall limb alignment in such cases, reducing the risk of patellar re-luxation [1–4, 6].

**Fig 1:**
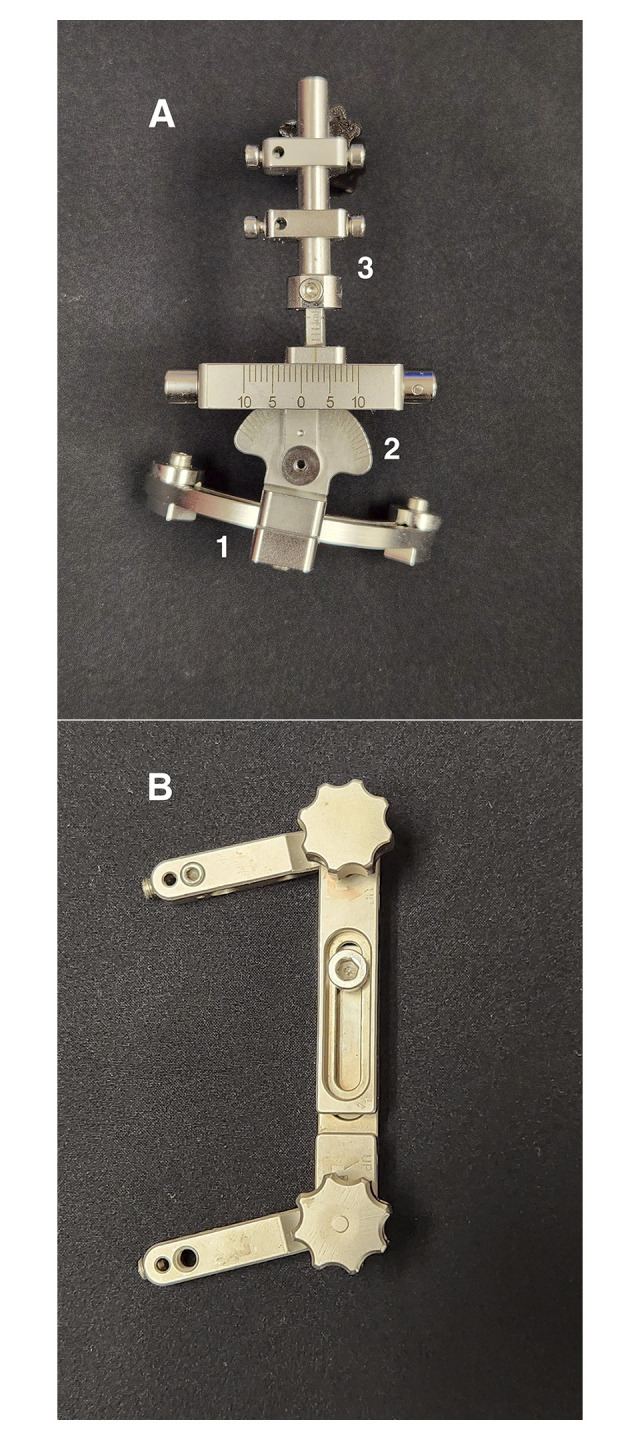
Mini deformity reduction device (DRD) jig and Mini Rail jig. **(A)** Mini deformity reduction device jig components: 1) Arch, 2) Hinge, 3) Rod, and (**B**) Mini Rail (Slocum) jig. Each component of the Mini DRD is movable independently of the others, allowing for greater precision and control of osteotomy manipulation in a three-dimensional plane.

Previously, the use of the Slocum tibial plateau leveling osteotomy (TPLO)-jig during femoral osteotomy has been described, serving as a temporary stabilizer of the osteotomy site [1, 2] (Fig 1). Its reported use suggests the need for additional stabilizers, such as bone reduction forceps or divergent Kirschner wires to achieve adequate stability and allow for the placement of a bone-plate [1, 9]. In addition to stability concerns, torsional correction performed with the Slocum jig is reported to result in a translational deformity, which must be further corrected visually to ensure appropriate limb alignment [1].

More recently, Panichi et al. (2016) have described the use of a novel deformity reduction device (DRD)-jig in the surgical management of patellar luxation (Fig 1) [1]. The DRD-jig is designed to serve as a hybrid, temporary external fixator for stabilizing the osteotomy, allowing for controlled realignment of bone segments and placement of a bone plate for rigid internal fixation [1, 9]. Unlike the Slocum TPLO-jig, the DRD-jig is stabilized by four pins, two above and two below the osteotomy site. These four pins ensure adequate stability and negate the need for additional stabilizers such as reduction forceps or divergent Kirshner wires. Following completion of the osteotomy with the DRD-jig in position, adjustment of individual micrometric screws associated with the hinge and the arch, along with the associated scales etched on the device, allows for controlled correction and temporary stabilization during the plating process.

Previous studies have compared the use of the Slocum-TPLO jig to the DRD Jig during distal femoral osteotomies with varying degrees of varus/valgus deformities [1, 9]. They have concluded that the DRD jig may be used during closing and opening wedge ostectomies to correct torsion-angulation deformities. The objective of the current study is to further investigate the use of the micro/mini DRD jig in comparison to the Slocum-TPLO jig for correction of varying degrees of torsional deformities of the distal femur in a model of medium-sized dogs when a set varus deformity of 15 degrees is present. We hypothesize that there will be no significant difference in the precision of correction of the torsional deformities between the two jig types.

## Materials and methods

### Bone model development

**Fig 2:**
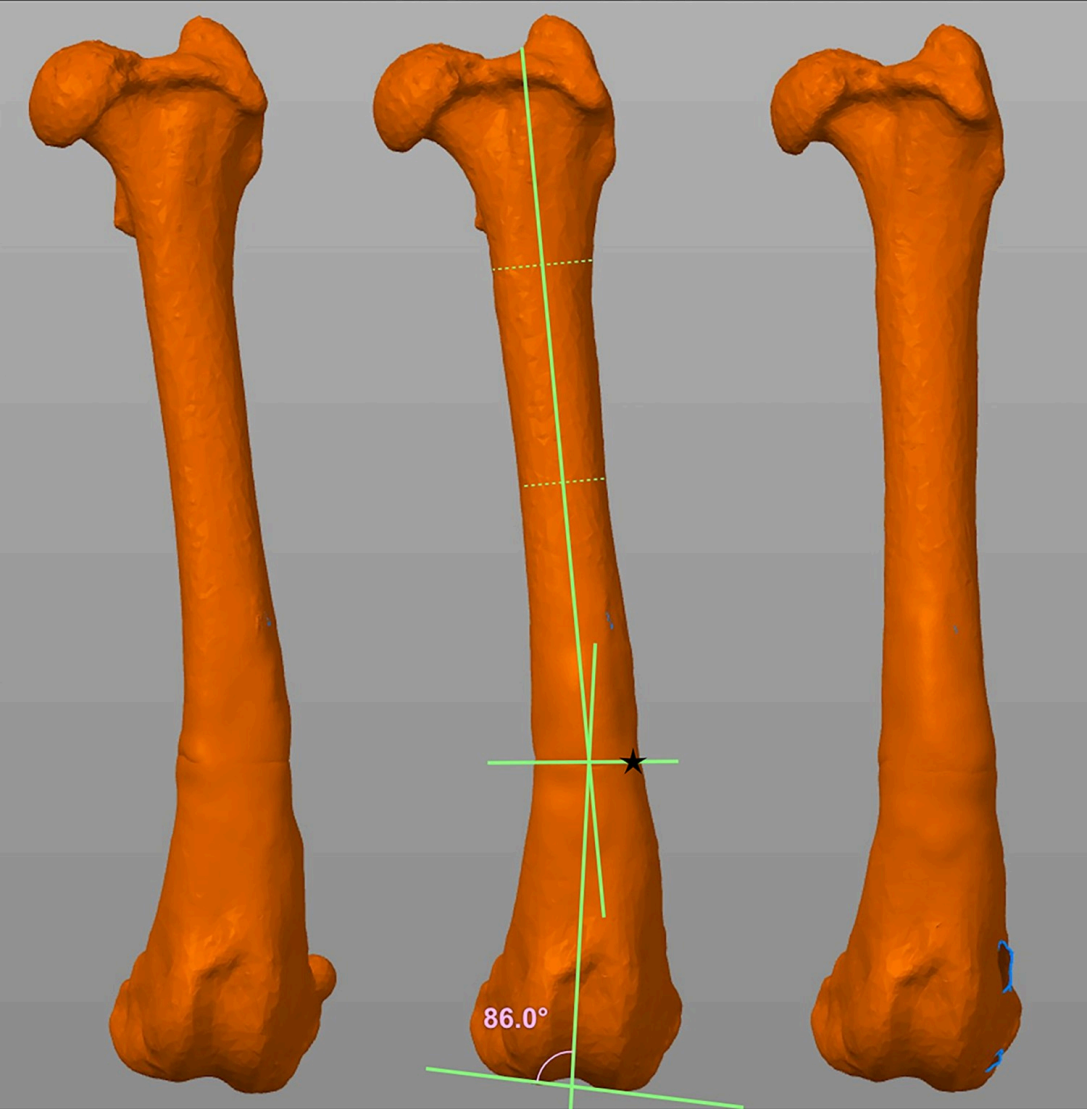
Frontal plane image of each deformity model with a standard 15 degree varus deformity. A) The 15 degree external torsion model (group 1). B) The 20 degree external torsion model (group 2) with pre-surgical planning using the center of rotation of angulation (CORA) methodology. C) The 30 degree external torsion model (group 3). Deformities applied to the models were created with Materialise Mimics and Meshmixer software.

**Fig 3:**
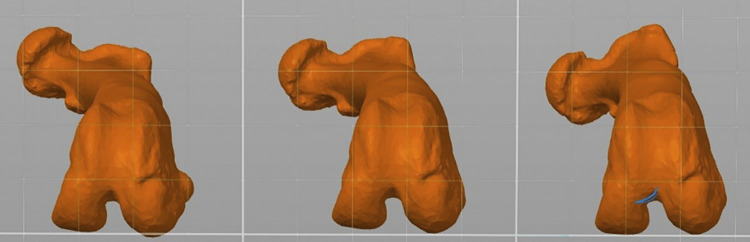
Axial plane image of each deformity model with a standard 15 degree varus deformity. A) The 15 degree external torsion model (group 1). B) The 20 degree external torsion model (group 2). C) The 30 degree external torsion model (group 3). Deformities applied to the models were created with Materialise Mimics and Meshmixer software.

The femoral models (n = 60) were developed based on an approximately 16.5 kg normal canine femur. A computed tomography (CT) scan of the normal canine femur was performed. Using Digital Imaging and Communications in Medicine (DICOM) software Horos, an.stl file was generated, and subsequently modified in Mimics (Materialise, Leuven, Belgium) to create a representative deformity model for each of the three different deformity groups. Image files were uploaded into Materialise Mimics and Meshmixer software (Autodesk Meshmixer (RRID:SCR_015736), enabling modulation of the 3D reconstructed femurs to apply the desired deformities. A standard varus deformity of 15 degrees was created in all models. Next, 15 degrees of external rotation were applied to the model, resulting in a representative 3D reconstruction for femurs included in Group 1. The same process was applied to create external torsional deformities of 20 degrees and 30 degrees, resulting in representative 3D reconstructions of femurs in Groups 2 and 3, respectively (Figs 2 and 3). Bone models were then printed in the 3D Prusa i3 printer (71 Prusa MK3S+) with a Polylactic Acid filament.

Once bone models were developed for each of the three deformity groups, they were further subdivided based on the jig-type to be used for correction. Bones to be corrected using the DRD jig were assigned to subgroup a, while those to be corrected using the Slocum jig were assigned to subgroup b (Table 1).

**Table 1:** Groupings based on deformity and jig type used.

	Varus Deformity Magnitude	External Torsional Deformity Magnitude	Jig Type Used	# of models
**Group 1a**	15 degrees	15 degrees	DJD	10
**Group 1b**	15 degrees	15 degrees	Slocum	10
**Group 2a**	15 degrees	20 degrees	DJD	10
**Group 2b**	15 degrees	20 degrees	Slocum	10
**Group 3a**	15 degrees	30 degrees	DJD	10
**Group 3b**	15 degrees	30 degrees	Slocum	10

### Pre-surgical planning

Pre-surgical planning was based on computerized 3D reconstructions of each deformity type as described above. Standard CORA methodology was used to obtain measurements and plan the correction on a single representative model for each of the three deformity groups (Fig 2) [10, 11]. A corrected anatomical lateral distal femoral angle (aLDFA) value of 94 degrees was used on all femurs and a femoral torsion angle (FTA) of 15 degrees internal rotation based on previously published values [1–3, 7, 8].

For correction of the varus deformity in the frontal plane, the location and magnitude of the CORA were measured and recorded [10, 11]. The transverse bisecting lines were determined based on these measurements, thereby determining the location and magnitude of the closing wedge. For each group, the FTA was then measured on the axial projection of the representative femur for assessment of the transverse plane [7, 12–15]. For correction of the torsional deformities, the amount of correction was converted from degrees to millimeters using the formula C = 2πr / 360˚, where r is radius of the circumference of the femur at the level of the osteotomy site, and the C is the arch length of the circle or radian. Using the circular formula α/360 = C/2πr and solving the equation having alfa as the angle of correction for torsion. This equation and calculation were used only for models in subgroups b, which underwent correction using the Slocum TPLO jig. Models in subgroups a, undergoing correction with the DRD jig did not require its use, as the arch component of the device is designed to correct for torsional deformity based on pre-operative determination of the magnitude of torsion.

### Bone model preparation and deformity correction

Each bone was assigned a random number using an online random number generator. This number was written on the caudal aspect of the femoral neck and served as a method to identify which group each bone belonged to during the outcome assessment. To decrease operator error and attempt to replicate identical deformity corrections within each of the three groups, the planned osteotomy location and dimensions were drawn directly on each model based on the predetermined location of the CORA. A perpendicular line was drawn along the cranial cortex of the model and across the proposed wedge osteotomy to represent a starting point from which torsion would be corrected.

**Fig 4:**
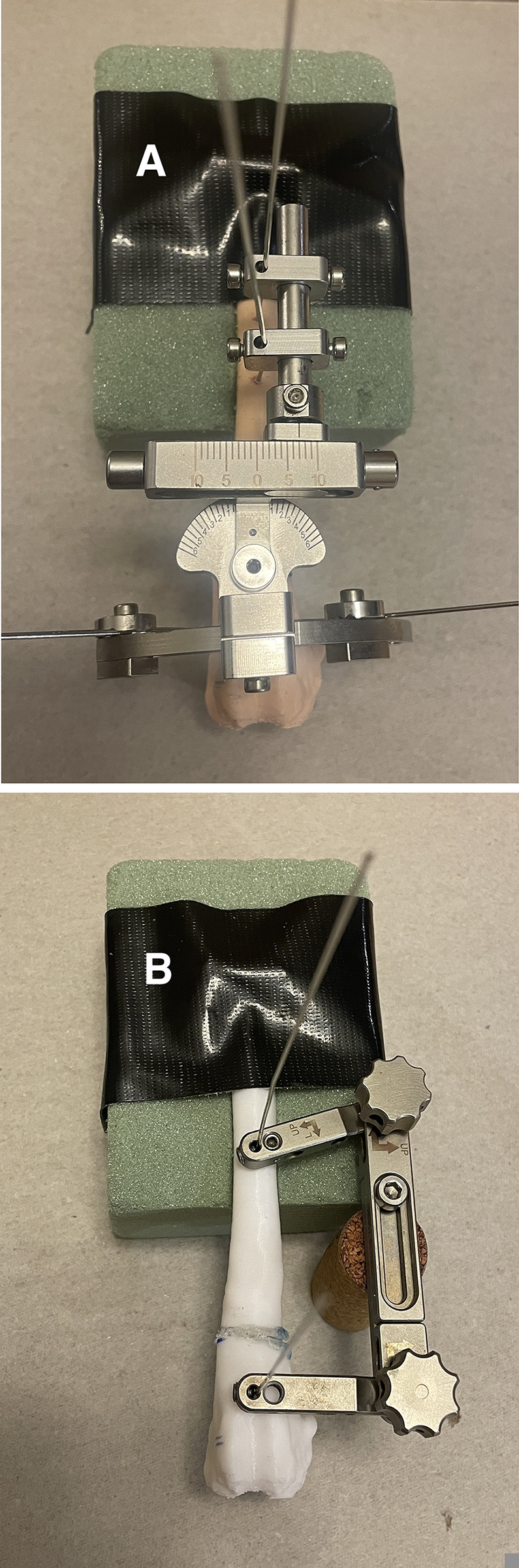
Bone model preparation with described coverage of the proximal femur. A) Post-correction of the DRD jig applied to the bone model. The arch allows for a total of 90˚ of rotation for torsional correction, and the hinge allows for a total of 120˚ of varus or valgus deformity correction. B) Post-correction of the Slocum jig applied to the bone model.

Next, a modeling clay was applied the proximal portion of the femur to mimic the visualization of a routine lateral surgical approach, while retaining the ability to palpate surgical landmarks such as the greater trochanter (Fig 4). Once each bone model was prepared as described above, the deformity correction was performed.

### Correction with the mini DRD jig

The mini DRD jig was applied to each model in similar fashion as described by Panichi et al., 2016 [1] (Fig 4). A 1.6mm Kirschner wire was positioned at the CORA in the craniocaudal plane. The cannulated hinge was placed over the CORA wire to establish the proximodistal alignment of the DRD-jig on the bone. Four Kirschner wires were then positioned. The two proximal pins were positioned craniocaudally in the proximal segment. Two additional pins were positioned craniomedially and craniolaterally in the distal segment. Following the placement of the four trans-fixation pins, the 1.6mm CORA wire was removed. Closing wedge osteotomies were performed with an oscillating sagittal saw. The wedge height was determined based on pre-correction planning. Following completion of the planned correction, all osteotomies were secured using a liquid adhesive.

### Correction with the Slocum TPLO jig

The Slocum-TPLO jig was applied to each model along the cranial aspect of the femur and secured with two pins as described by Brower et al, 2017 [2] (Fig 4). The distal pin was positioned in the sagittal plane, immediately proximal to, and parallel with the trochlear groove. The proximal pin was also placed in the sagittal plane, within the mid-diaphyseal region of the bone. The frame of the jig was positioned medially to allow access to the lateral aspect of the bone. Closing wedge osteotomies were then performed with an oscillating sagittal saw. The wedge height was determined based on pre-correction planning. Correction of the torsional deformity was accomplished by bending the distal jig pin to align two previously marked points along the osteotomy to achieve a post-correction FTA of 15 degrees. Following completion of the planned correction, two Kirschner wires were placed distolateral to proximomedial and distomedial to proximolateral to stabilize the post-correction osteotomy site. All osteotomies were secured using a liquid adhesive.

### Post-correction evaluation

Following fixation with the liquid adhesive, the jigs and modeling clay were removed. A standardized positioning guide was used during the post-correction assessment (Fig 4). Positioning of the bones were based on the previously reported acceptable standards for obtaining images of the femur [7, 8, 14–18]. The post-correction aLDFA and FTA measurements were obtained and recorded by a single investigator. The aLDFA served as the metric used to assess precision and accuracy of deformity correction in the frontal plane, while the FTA determined precision and accuracy of torsional deformity correction in the axial plane.

## Statistical analysis

The normality of the data was evaluated using the Shapiro‐Wilk test and QQ plot. Because of the validity of the normality, the mean and standard deviation were reported. Two sample t-tests were used to compare the difference in correction of torsional and varus deformities between the Slocum and DRD jig types at varying degrees of applied torsion. The one-sample t-test was used to compare if the post-correction torsion was significantly different from the ideal 15 degrees of internal rotation, and if the post-correction aLDFA was significantly different from the ideal 94 degrees. SAS, version 9.4, release TS1M8, was used for all analyses (SAS Institute Inc., Cary, NC, USA.). P <0.05 was considered significant.

## Results

Data obtained through this study allowed for the comparison of precision and accuracy between jig types used, as well as comparison of each post-correction measurement to reported normal values (Table 2). Correction of external torsion between the use of the DRD jig and the Slocum jig in groups 1a and 1b, and groups 2a and 2b were significantly different (P = 0.026 and P = 0.046, respectively). The correction of torsion was not significantly different between the two jig types in groups 3a and 3b (P = 0.018). When compared to the ideal torsional correction to 15 degrees of internal rotation, groups 1a, 1b, and 2b were significantly different (P = 0.0012, P = 0.001, and P = 0.005 respectively) from ideal. The post-correction torsion angle for groups 2a, 3a, and 3b were not significantly different from the ideal 15 degrees of internal rotation.

**Table 2:** Data summarizing post-correction FTA and aLDFA measurements for each group and respective jig type used for deformity correction, where subgroups a utilized the DRD jig, and subgroups b utilized the Slocum jig. P-values are included for the comparison of jig type used within each of the 3 primary groups, where P-value < 0.05 is considered significant.

Group	Post-Correction FTA		Post- Correction aLDFA	
	Mean ± SD	DRD vs. Slocum P-value	Mean ± SD	DRD vs. Slocum P-value
**1a**	16.89 ± 1.28	0.026	91.02 ± 2.86	0.993
**1b**	19.01 ± 2.44		91.01 ± 2.31	
**2a**	15.85 ± 1.64	0.046	89.16 ± 2.21	0.575
**2b**	17.88 ± 2.5		89.78 ± 2.63	
**3a**	15.05 ± 2.08	0.183	88.69 ± 1.39	0.872
**3b**	16.58 ± 2.81		88.9 ± 3.79	

*Footnote*: Data reported for post-correction FTA and aLDFA are mean and standard deviation in degrees. Abbreviation: FTA, femoral torsion angle; aLDFA, anatomic lateral distal femoral angle.

**Fig 5:**
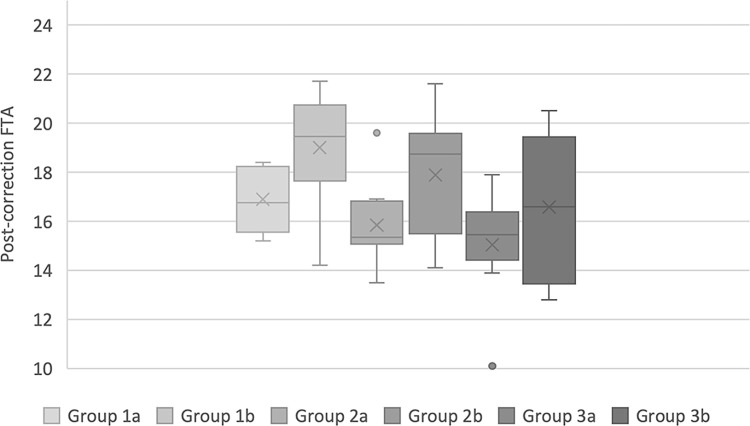
Box plot of post-correction FTA where subgroup a have been corrected with the DRD jig, and subgroup b with the Slocum jig.

The mean post-correction FTA results of subgroups a, whose models were corrected using the DRD Jig, were consistently closer to the ideal post-correction value compared to those in subgroups b, although these differences were not always statistically significant (Fig 5). (Group 1a - 16.89 ± 1.28, Group 1b 19.01 ± 2.44, Group 2a - 15.85 ± 1.64, Group 2b 17.88 ± 2.5, Group 3a – 15.05 ± 2.08, Group 3b 16.58 ± 2.81). In all groups, the standard deviation from mean post-correction FTA value was greater when using the Slocum-TPLO jig, indicating greater variability and less accuracy of correction within the subgroup.

**Fig 6:**
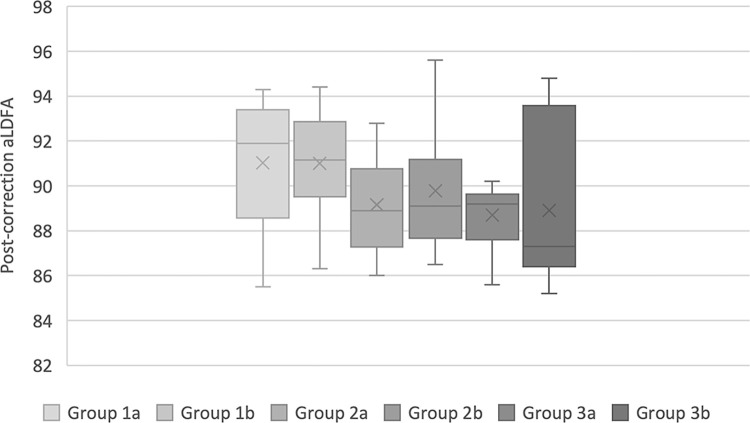
Box plot of post-correction aLDFA where subgroup a have been corrected with the DRD jig, and subgroup b with the Slocum jig.

For the correction of varus deformity in the frontal plane, no significant difference was found between the two jig types in groups 1, 2, or 3 (Fig 6). However, correction of the varus deformity in the frontal plane was significantly different from the ideal aLDFA of 94 degrees in all groups (P<0.05).

## Discussion

With the goal of restoration of anatomical alignment, the use of jigs during corrective osteotomies have reported benefits of temporary stabilization and increased precision and predictability of the repair [1, 2, 9]. The two jig types used in this study, the mini deformity reduction device (DRD) jig and Slocum jig, approach this task differently, prompting the question of whether one would outperform the other when precision of the correction was evaluated. To the author’s knowledge, this is the first report of the use of the mini DRD jig during corrective distal femoral osteotomy. Accurate placement of both jig types was essential for the successful use of each device. To decrease inter-operator variability, a single investigator (SS) performed all jig placements and corrections on the 60 models.

The use of each jig provided a unique set of challenges during their application to the bone model and in providing stability to the osteotomy site. The mini DRD jig requires the placement of 4 Kirschner wires to stabilize the jig and bone. The size of this jig in comparison to that of the femur models used in this study of a 16.5kg dog made its application challenging with little room for error during placement. Once positioned, the DRD jig covered a majority of the bone, making positioning of the saw during the osteotomy challenging. Despite this, the mini DRD jig provided adequate stability of the osteotomy site and maintained the fragments in reduction throughout the correction. The use of the Slocum TPLO jig during the distal femoral osteotomy provided little stability and required the use of additional instruments and Kirschner wires to stabilize the osteotomy during correction. Completion of the osteotomy was easier with the Slocum jig, as this device did not interfere spatially with the corrective procedure.

The use of the mini DRD jig negates the need for the mathematic calculation of torsional correction using the equation C = 2πr / 360˚. This equation is not representative of the circumference of the femur, as the bone itself is not a 360˚ circular structure, an assumption made by the formula for geometric arc and chord length. As such, an inherent error is present with this equation during the correction of torsion with the Slocum jig. The mini DRD jig is pre-set with the deformity present based on presurgical planning and applied to the malformed bone. Once secured with the 4 stabilizing Kirschner wires, the osteotomy can be performed. The dials and scales on the device are then used to precisely correct the deformities back to the zero or neutral mark. For each of the bone models, following completion of the osteotomy, the dial on the hinge was first adjusted to correct the varus deformity from the pre-set 15 degrees to the neutral 0 degree mark. Next, the dial on the arch was adjusted to correct the varying degrees of external torsion between the three groups. This process standardized correction of the femoral deformities between all models in subgroups a (those undergoing repair with the DRD jig).

Correction using the Slocum jig provided less standardization of the correction, less stabilization of the osteotomy site post correction, and relied on the mathematic equation C = 2πr / 360˚ for correction of external torsion. Once the magnitude of torsional correction was calculated for each of the three subgroups utilizing the Slocum jig, marks were created on each model to identify the degree and magnitude of rotation needed to restore the femoral torsion angle to 15 degrees of internal rotation. Due to a lack of inherent stability with the Slocum jig alone, additional stabilizers in the form of divergent Kirschner wires and point-to-point bone holding forceps were required. Spatially, the size of the models representing a 16.5kg dog made placement of these additional stabilizers challenging.

Based on the results of this study, the DRD jig has demonstrated superior ability to facilitate more accurate and precise correction of torsional deformity. Post-correction FTA assessment between the two jigs consistently resulted in a more precise and accurate correction using the DRD jig, despite not always being significant. The increased variance observed within subgroups designated as b may be attributed to a less stable temporary fixation with the Slocum-TPLO jig. Furthermore, the correction of torsion in the distal femur, a structure that is not perfectly circular, relied on a mathematical equation for geometric arc and chord length. This approach may have contributed to the observed variance. Post-correction FTA assessment also indicates that when the magnitude of deformity was less than 30 degrees of external rotation, there was a statistically significant difference between the two jig types. This is assumed to be the result of challenges associated with accuracy and precision when dealing with magnitudes of lesser value and the associated narrower margin for error.

In many cases, it is recommended to visually assess the correction to ensure appropriate alignment. This however can be challenging when viewing the clinical case, as many anatomic landmarks are not readily accessible or viewed. Additionally, reliance on surgeon’s experience to aid in this visual component of the correction is something that cannot be standardized, and as such, experience remains an important factor when evaluating accuracy and precision of corrective procedures.

There is a lack of statistical difference for correction of the varus deformity in the frontal plane between the two jig types. The significant variance from the ideal aLDFA of 94 degrees for all six groups inaccurate execution of the corrective osteotomy. CORA methodology was used for pre-operative planning in all groups [10, 11]. The CORA was determined to be 33mm from the distal joint margin, and the height of the wedge to be removed was 14mm. For all groups, the deformity in the frontal plane was overcorrected, with the mean value for post-correction aLDFA ranging from 88.69 to 91.02 between all groups. Factors that may have contributed to this include the width of the saw blade in relation to the planned osteotomy drawn on each model, and thermal effects of a high-speed saw on the polylactic acid filament used to print the models.

The size of the bone models used in this study presented a unique challenge in that small changes during correction resulted in larger magnitudes of variation and error in comparison to what may be expected when using a larger model. This may be another consideration for the lack of accuracy of the correction of varus deformity. Despite the statistically significant variation from the ideal aLDFA of 94 degrees, clinical significance remains unclear.

A key feature of this study that sets it apart from similar assessments of deformity correction is the use of a modeling clay to cover surgical landmarks during the correction. Placement of a thick layer of clay over the proximal 1/3 of each femur, and the positioning of the proximal femur within a block structure to elevate the bone prior to correction aimed to mimic the in vivo positioning and exposure of the distal femur and prevented investigators from visually assessing each bone prior to completion of the planned corrective osteotomy. The modeling clay allowed for palpation of the greater trochanter, as would be routinely palpable in surgery, but prevented visual assessment for confirmation of accuracy during correction. In an attempt to limit the impact of surgical experience on correction, pre-surgical plans were tightly adhered to, with no allotment for additional visual correction once the osteotomy was performed. While this approach is useful in objectively comparing the accuracy and precision of the two jig types, it does not accurately represent the approach to correction in clinical patients, where visual assessment and critical intraoperative evaluation is recommended.

There are several additional limitations and potential sources of error to be discussed. First, differences in precision of osteotomies due to variations and/or errors in saw handling may impact overall accuracy and precision of the correction. While the use of 3D printed bone models allowed for standardization of deformities between all samples within a group and provided an easily obtainable sample size for the study, limitations to the use of such models inherently exist, as they may not precisely mimic conditions encountered in vivo. The lack of surrounding musculature and soft tissue structures prevents an accurate assessment of ease of use of each of the jig types and does not accurately allow for the assessment of the known impact of these surrounding structures on overall deformity correction.

As is the case with many surgical procedures, surgeon experience is a factor to consider. In the current study, multiple steps were taken in an attempt to limit the impact of surgeon experience. CORA methodology was utilized to plan the desired correction for each of the 6 groups. Pre-operative planning was performed by authors CF, with over 15 years of experience, and SS, a surgical resident in training. Following the development of the surgical plan, each of the 60 bones were marked by a single investigator (SS) to standardize the correction. All corrections were then performed by a single investigator (SS), and deviation from the plan was not permitted.

## Conclusions

Jig selection during distal femoral osteotomy for the correction of torsional deformity may result in significantly different post-correction alignments when the magnitude of external torsion is less than 30 degrees. Clinical significance of this variance from the aLDFA value of 94 degrees and FTA value of 15 degrees remains unclear, as reported normal ranges vary, and a single value has not been established. Intra-operative visual assessment of alignment should be implemented to guide corrections.
